# The Behavior of Superabsorbent Polymers (SAPs) in Cement Mixtures with Glass Powders as Supplementary Cementitious Materials

**DOI:** 10.3390/ma12213597

**Published:** 2019-11-01

**Authors:** Khashayar Farzanian, Babak Vafaei, Ali Ghahremaninezhad

**Affiliations:** Department of Civil, Architectural and Environmental Engineering, University of Miami, Coral Gables, FL 33146, USAbxv158@miami.edu (B.V.)

**Keywords:** SAPs, capillary forces, desorption, cementitious materials, glass powders

## Abstract

The absorption and desorption of superabsorbent polymers (SAPs) in cement mixtures containing two different glass powders as supplementary cementitious materials are examined in this paper. Two SAPs with different chemical compositions were synthesized in-house and used in the experiments. SAP absorption was investigated directly through the mass change of SAPs in cement slurries, as well as indirectly using the flow test. Scanning electron microscopy was used to monitor the desorption of SAPs using samples prepared with freeze-drying. Hydration and setting time were evaluated to explain the desorption behavior of SAPs. SAP absorption generally increased in pastes with glass powders. The desorption rate of SAPs in different pastes was shown to correlate with the onset of solid skeleton development in the pastes. The addition of SAPs reduced autogenous shrinkage in neat cement paste more than in pastes with glass powders.

## 1. Introduction

Cementitious materials with low water/binder can be susceptible to autogenous shrinkage cracking [1]. Once cracks occur in cementitious materials, they increase the transport rate of harmful agents into the material, facilitating and accelerating other chemical and physical degradation processes in the materials [2,3]. Thus, autogenous shrinkage cracking is a critical durability issue in cementitious materials characterized with low water/binder [4]. In mixtures with low water/binder, self-desiccation leads to a reduction in relative humidity, creation of menisci at the air–pore solution interface, and development of the capillary forces in the pore solution [5,6]. The capillary forces pull the solid skeleton inward leading to an overall reduction in the volume of the material. If the material is constrained against volume changes, tensile stresses are developed and cracking occurs when the tensile strength of the material is reached. 

The supply of water into the interior of materials to maintain relative humidity and mitigate the negative consequences of autogenous shrinkage is referred to as internal curing [6,7,8,9,10,11,12,13,14,15,16,17,18,19]. Superabsorbent polymers (SAPs) are one class of materials used for internal curing that have been demonstrated to be effective in mitigating autogenous shrinkage [6,12,13,14,15,16,19]. The ability to control the physical and chemical properties of SAPs to adapt to desired applications in cementitious materials is advantageous compared to other internal curing materials such as saturated lightweight aggregates. 

The provision of additional water from SAPs has been shown to improve hydration in cementitious materials [14,20,21]. Prior investigations have examined the influence of SAPs on the transport characteristics of cementitious materials [13,14,22]. It was found that the addition of SAPs could reduce chloride penetration [13,14]. Improvements in microstructure due to addition of SAPs have also been indicated by other investigators [16,21,23,24,25]. De Meyst et al. [26] performed a parametric study to investigate the effect of different degrees of cross-linking, particle sizes, and amounts of solubles of SAPs on their absorption and their influence on mortar properties. Additionally, other benefits including enhanced resistance to freeze–thaw induced degradation [27] and self-healing potential [28,29,30] of SAPs have been discussed in the literature. Tan et al. [31] examined the effect of two kinds of dosing methods—dry and swollen SAPs—on the properties of mortars and showed that both dosing methods improve the freeze–thaw resistance of mortars. Woyciechowski et al. [32] studied how dosing methods and material characteristics of SAPs affect the internal curing of concrete. Despite the above-mentioned benefits, use of SAPs typically reduces compressive strength, primarily due to the formation of macrovoids left in the microstructure after SAPs desorb [16,33,34,35,36]. The nature of the influence of SAPs on the properties of cementitious materials depends on the mix formulation of cementitious materials, as well as the physical and chemical characteristics of SAPs [1,13,15,34,36]. 

Nowadays, most concrete structures include supplementary cementitious materials in their material formulation to improve their life cycle [37]. Slag [38,39,40,41], fly ash [42,43,44,45], silica fume [46,47,48], glass powder [49,50,51,52,53,54,55,56,57,58], and metakaolin [59,60] as supplementary cementitious materials have been examined in the prior investigations. The use of SAPs in mixtures containing supplementary cementitious materials including slag [13,61,62], fly ash [24,62], glass powders [51], and silica fume [13,15] has been studied in a few previous investigations. Knowledge of the behavior of SAPs, including SAP absorption and desorption, in cementitious materials is critical for the effective application of internal curing. The dependence of SAP absorption on its chemical and physical characteristics in different ionic solutions was the subject of several prior efforts [30,63,64,65,66,67,68,69,70,71]. However, these efforts primarily examined the behavior of SAPs in a solution medium related to only Portland cement. 

Factors affecting the desorption process of SAPs include capillary forces in the pore structure and the chemistry of pore solution, which evolve with hydration in cementitious materials [33,72]. Although SAP desorption in a solution medium or in air was explored by a few researchers [73,74], the effect of capillarity developed in the pore structure on the desorption of SAPs has received very limited attention [22,75,76,77,78]. Neutron tomography [76] and neutron radiography [78] were employed to probe the desorption of SAPs in cement paste. The nuclear magnetic resonance (NMR) technique was adopted to investigate the water release from SAPs in a cementitious matrix [79,80]. Our previous studies provided insight into the influence of capillarity on the desorption of SAPs in contact with a porous cementitious material [22,75]. Despite previous studies, detailed desorption of SAPs in cementitious materials with supplementary cementitious materials is scarce. A recent paper by our group discussed the interaction between SAPs and cementitious materials containing fly ash, slag, or silica fume [81]. This paper aims to investigate the interaction between SAPs and cement mixtures containing two types of glass powder as supplementary cementitious materials. A novelty of the current work is the direct temporal tracking of the desorption of SAPs in cement mixtures containing glass powders using scanning electron microscopy. The absorption of SAPs in different slurries was evaluated based on a direct mass change measurement, as well as an indirect method based on a workability measure. The heat of hydration and setting time were measured to help explain the desorption of SAPs in different pastes. Fourier transform infrared spectroscopy (FTIR) was employed to probe the chemical changes on the surface of SAPs. The autogenous shrinkage measurement was also carried out.

## 2. Experimental Work

### 2.1. Materials

#### 2.1.1. Superabsorbent Polymers (SAPs)

SAPs used in this study were cross-linked poly(sodium acrylate-co-acrylamide) hydrogels. The SAPs were synthesized using free radical polymerization, as detailed in our prior works and by others [22,82,83]. Two types of SAPs, S-A and S-B, with different chemical compositions were used in the experiments to allow us to study the effect of chemical composition on SAP behavior in cementitious mixes. Table 1 provides the amount of different chemicals used in the synthesis of SAPs. Acrylic acid was first dissolved in distilled water, and sodium hydroxide solution (13.5%) was used to partially neutralize acrylic acid. Acrylamide and the cross-linker (*N,N’*-methylenebisacrylamide (MBA)) were added to the solution and subjected to stirring for half an hour. The degasification of the solution was performed via bubbling argon into the solution. Next, the polymerization initiator (ammonium persulfate) was introduced into the solution. The solution was transferred into two parallel glass plates and allowed to gelate in an oven at 60 °C for 3 h. The distance between the two glass plates could be changed to vary the thickness of SAP layers. Then, the SAPs were taken out of the glass plates and their surface was gently washed with isopropanol to eliminate the unreacted chemicals. Then, the SAPs were immersed in distilled water for 3 h and then left outside of distilled water overnight. Next day, the disks were punched from SAP layers and placed in an oven to dry at a temperature of 60 °C. Thin disks (dry thickness: 0.25–0.28 mm) to be used in the absorption test and thick disks (dry thickness: 1.6 mm) to be used in the FTIR analysis were prepared. The dry diameter of the disks was the same and equal to 9 mm. In order to examine the desorption behavior of SAPs in pastes, microstrips (dry cross section: 0.25–0.28 mm) were cut using a blade. SAPs, in the form of powder to be used in the autogenous shrinkage experiment, were also prepared by grinding dried SAP layers in a coffee grinder and sieving them to obtain SAP powders with a size in the range of 75 to 425 µm. This size range was selected because it is within the size range of SAP particles used in the practical applications.

#### 2.1.2. Binders

The cement used in this study was a type I/II portland cement. Two types of glass powder with different chemical compositions, denoted as G1 and G2, were used. G1 is derived from post-consumer waste glass, and G2 is an amorphous aluminosilicate material manufactured from waste glass fibers. Table 2 provides the chemical properties of these materials. The mean particle size of G1 and G2 was ~8.4 µm. The effect of G1 and G2 as supplementary cementitious materials have been studied in our previous work [84]. The mix design of the pastes and slurries is shown in Table 3. Pastes with 100% cement, denoted as C, and pastes with 40% replacement of cement with G1 and G2, denoted as C-G1 and C-G2, respectively, were mixed. The effective water/binder of pastes was 0.3, except for slurries to be used in the absorption experiment, where water/binder was 2. To improve workability, a lignosulfonate-based super plasticizer in the amount of 0.5% of the mass of the binder was used in all pastes and slurries.

### 2.2. Experimental Investigations

#### 2.2.1. Absorption

In this study, the absorption of S-A and S-B in cement slurries with and without glass powders was measured. In most prior studies, the absorption of SAPs was evaluated in a solution medium obtained through filtration of cement mixtures or synthesized using chemical compounds [30,63,64,68,69,70]. One shortcoming of using a solution medium for absorption measurement is that it neglects the possible chemical and physical effects arising from the interaction between SAPs and reacting binder solid particles in the mixture. Therefore, in this study, SAP absorption was investigated in different slurries with a water/binder of 2. The slurries were kept sealed in a 100 mL tube to minimize exposure to air and potential carbonation. The slurries were routinely agitated to maintain uniform dispersion. The absorption of SAPs was obtained by measuring the change in the mass of SAPs at different times using a balance.

#### 2.2.2. Flow Test

Use of the flow test to estimate the SAP absorption has been attempted in previous investigations [36,85,86]. In this method, the SAP absorption is assumed to be equal to the additional water required to maintain the same flow value when SAPs are added to the paste. The flow test was performed on pastes with a water/binder of 0.3. In pastes with SAPs, the water/binder ratio was 0.35 and the dosage of SAPs was varied to obtain the same flow value as the pastes without SAPs. The additional water/binder of 0.05 is adopted, as, in theory, this amount is required to mitigate autogenous shrinkage in pastes with water/binder of 0.3 [6]. In the flow test, pastes were poured into a stainless steel cone (bottom diameter: 10 cm; top diameter: 7 cm; and height: 5 cm) and placed on a table (ASTM C1437-15). The paste was allowed to rest for 10 min; then, the cone was lifted and the table was dropped 25 times at a constant frequency over a period of 15 s. The two perpendicular bottom diameters of the paste were measured and the average is reported as the flow value.

#### 2.2.3. FTIR Analysis

This analytical technique was adopted to probe the chemical effects on SAPs as a result of interaction with different slurries with and without glass powders. Thick SAP disks were utilized for the FTIR analysis. SAP disks were submerged in different slurries for 8 h and then removed and their surface cleaned with isopropanol. The disks were then directly inserted into the FTIR instrument (PerkinElmer Paragon 1000, Perkin-Elmer, Waltham, MA, USA). 

#### 2.2.4. Heat of Hydration

The hydration of different pastes was studied using isothermal calorimetry (ASTM C1702). Glass ampoules were filled with approximately 6 g of pastes and sealed to protect them against moisture loss. The glass ampoules were placed in a TA Instrument isothermal calorimeter to measure the heat flow. Prior to data collection, the instrument was preconditioned at a temperature of 23 °C.

#### 2.2.5. Desorption of SAPs in Pastes

The desorption of SAPs embedded in different pastes was studied via imaging the change in the cross sectional dimensions of SAP microstrips at different times during the early hydration of the pastes. Polypropylene tubes were cut open in the axial direction, and their bottom half was filled with pastes. A SAP microstrip was placed on the paste parallel to the axis of the tube and the top half of the tube was filled with paste and then the entire tube was sealed. To image the SAPs, the tubes were submerged in liquid nitrogen to stop hydration, and then cut perpendicular to expose the cross section and vacuum-dried in a lyophilizer. The sections were then examined using a JEOL (JSM-6010PLUS/LA, JOEL, Tokyo, Japan) SEM. Water loss from SAPs was estimated as 1−*S*_t_/*S*_0_, where *S*_t_ is the cross sectional area of SAP microstrip at time t and *S*_0_ is the cross sectional area of the macrovoid corresponding to the largest absorption of SAPs in the paste. More than five replicates were imaged and analyzed to account for statistical variability. Sections of the pastes away from the SAPs were polished and prepared for SEM imaging following the procedure detailed in [35].

#### 2.2.6. Autogenous Shrinkage

The autogenous shrinkage of pastes was measured according to ASTM C1698-16. In this experiment, pastes were poured into corrugated polyethylene tubes, and lids were used to seal both ends of the tubes. The variation in the length of the tubes was monitored using a DC linear variable differential transformer (LVDT). The measurement began at a time coinciding with the final setting time of the pastes. Specimens with and without S-B were prepared. A minimum of two specimens were employed for each paste. 

## 3. Results and discussion

### 3.1. Absorption and Flow Results

**Figure 1:**
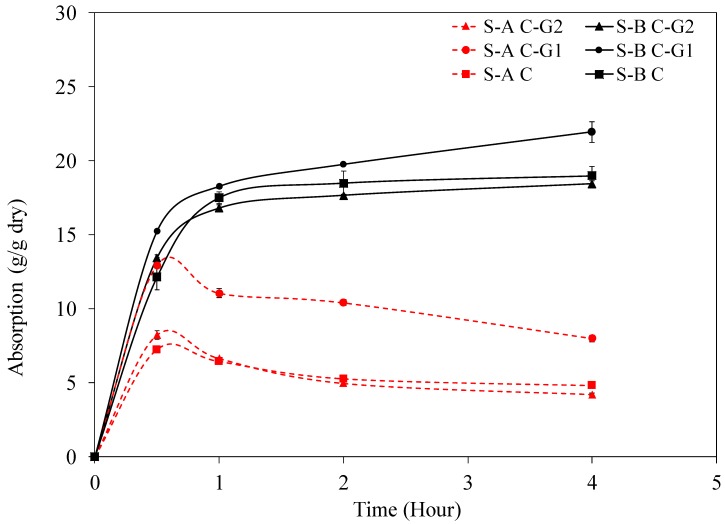
Absorption behavior of S-A and S-B in neat cement slurry, C, and in slurries with glass. powders, C-G1 and C-G2.

The absorption behavior of the two SAPs with different chemical compositions, S-A and S-B, in different slurries—C, C-G1, and C-G2—is illustrated in Figure 1. As detailed in the Experimental section, C, C-G1, and C-G2 slurries refer to the slurries made of neat cement and cement with glass powders G1 and G2, respectively. The data corresponding to S-A C and S-B C are reproduced from [81]. Note that S-A and S-B showed a significantly lower absorption in slurries compared to their absorption in distilled water, which was approximately 130 (g/g) and 85 (g/g), respectively. The absorption measurement in distilled water was conducted using SAP powder. The lower absorption in slurries is due to the presence of cations, such as Na^+^, K^+^, and Ca^2+^, in the slurries [30,68,71]. These cations are released into the slurries because of solid binder particle dissolution. In the presence of these cations, repulsive forces between negatively charged carboxylate groups in the SAP network are reduced through the screening effect; additionally, divalent cations, such as Ca^2+^, can form complexes with the negatively charged carboxylate groups. The screening effect and complex formation lead to a reduction in the swelling of the SAPs [30,68,71]. Unlike in distilled water, S-A exhibited a lower absorption compared to S-B in slurries, which could be attributed to a higher concentration of carboxylate groups from acrylic acid in S-A than in S-B, which provides more screening effect and complexation. Note that the absorption of SAPs in C-G1 slurry is more than in C and C-G2 slurries, and the difference is more pronounced in S-A than S-B, as seen from Figure 1. 

The percentages of S-A and S-B in different pastes obtained from the flow test are listed in Table 4. As mentioned previously, these percentages were obtained based on achieving the same flow value for the pastes with a water/binder of 0.35 containing the SAPs as the respective pastes with a water/binder of 0.3 without SAPs. The flow value of C, C-G1, and C-G2 without SAPs was similar at 20 cm. The absorption capacity of S-A and S-B is higher in C-G1 than C-G2; this is seen from the lower SAP percentage in C-G1 than in C-G2. This observation is in agreement with the slurry absorption results, as shown in Figure 1. SAP percentages in the neat cement paste were higher than in the pastes containing glass powders, which indicate lower absorption in the neat paste than in the pastes containing glass powders. One possible explanation for this could be a lower ionic concentration of the pore solution in the paste due to a 40% mass replacement of cement with glass powders G1 and G2. Although, the effect of cement replacement on SAP absorption in C-G1 slurry was evident, no significant change was noticed in the case of C-G2 slurry. A comprehensive chemical composition analysis of the solution phase of the slurry and paste is needed to provide an understanding of the underlying reason for such a behavior and this will be reported in the future contributions. Note that the chemical and physical interactions between the SAPs and solid binder particles could also affect the absorption of the SAPs and could play a role in the observed absorption behavior of SAPs in the neat cement paste and the pastes with glass powders. 

### 3.2. FTIR Analysis

**Figure 2:**
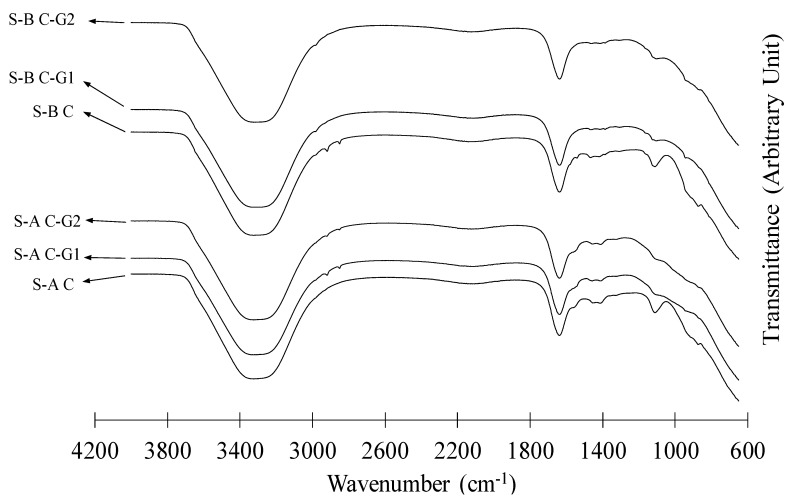
FTIR spectra of S-A and S-B after absorption in different slurries.

The FTIR spectra of SAPs after absorption in different slurries, C, C-G1, and C-G2 are depicted in Figure 2. The data corresponding to S-A C and S-B C are reproduced from data in [81]. The spectra of SAPs exhibited similar features: the peak at 1638 cm^−1^ [87] is attributed to amide groups, and the peaks at 1560 cm^−1^ and 1408 cm^−1^ are associated with carboxylate groups [30,74,88]. A broad peak at about 3300 cm^−1^ is assigned to the O–H bond of free water in the SAPs [89]. However, a distinguishing feature can be noticed in peaks at approximately 1110 cm^−1^ and 874 cm^−1^, which correspond to the carbonate groups. These peaks appear to be stronger in both S-A and S-B after absorption in C slurry than in C-G1 and C-G2 slurries. The stronger peaks could indicate a more pronounced formation of carbonate compounds more likely calcium carbonate on the surface of the SAPs. Calcium carbonate is most likely a product of the carbonation of complexes involving Ca^2+^ and anionic groups of the SAP polymeric network. The formation of a whitish thin layer on the surface of SAPs has previously been noted in other studies [22,90]. 

### 3.3. Isothermal, Setting Time and SEM Imaging

**Figure 3:**
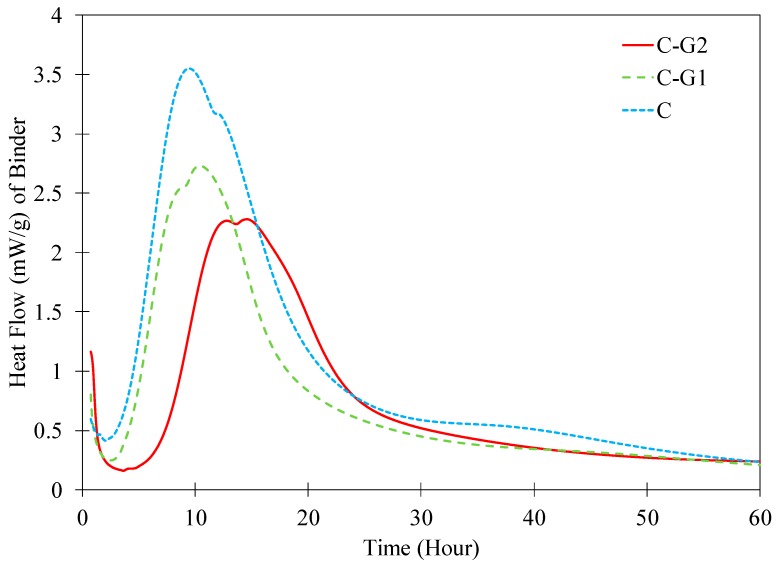
Heat flow of C, C-G1, and C-G2 pastes.

The hydration heat flow of the cement paste and the pastes with G1 and G2 with a 40% replacement rate obtained from isothermal calorimetry is demonstrated in Figure 3. All pastes showed a first peak at the very beginning corresponding to the dissolution stage of hydration. A second peak associated with the binding phase formation in the microstructure is seen in all pastes. This peak is higher in C paste than in C-G1 and C-G2 pastes. This can be attributed to the dilution effect as a result of cement replacement with glass powders. The peak in C-G1 paste seems to be slightly higher than that of C-G2 paste. A delay in the peak in C-G2 paste compared to C and C-G1 pastes is noticed indicating a slower hydration in C-G2 paste compared to the other two pastes. 

The initial and final setting times of different pastes are given in Table 5. Note that initial setting occurred first in C-G1, followed by C paste and C-G2 paste. The final setting time of C-G2 occurred later than that of C and C-G1 pastes. The longer setting time of C-G2 compared to other two pastes is in agreement with the observation made of the isothermal calorimetry results where the second peak in C-G2 was delayed compared to other pastes at early age. The final setting time is typically correlated with the onset of solid skeleton development and creation of capillary forces in the pores when the relative humidity decreases as a result of self-desiccation [78]. 

**Figure 4:**
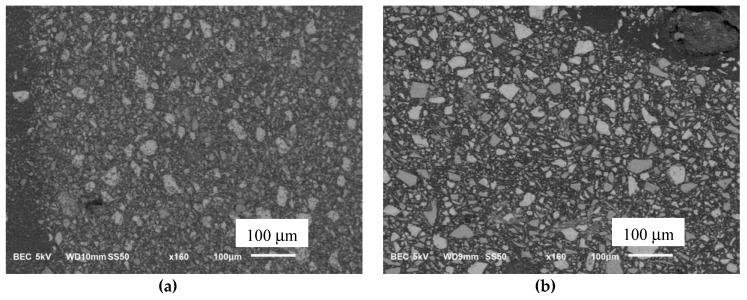
SEM micrographs of (**a**) C-G1 paste and (**b**) C-G2 paste at the age of 24 h.

The SEM micrographs in the backscatter mode of C-G1 and C-G2 pastes at the age of 24 h are shown in Figure 4. The solid portion of the microstructure consists of hydration products and unhydrated binders; bright regions corresponding to the unhydrated binders can be distinguished from the grayish regions corresponding to the hydration product. The backscatter mode of SEM allows for such a distinction of different phases based on brightness intensity. The unhydrated binders are seen brighter than other phases including hydration product and pores due to their higher average atomic number. A comparison of the two micrographs indicates a larger area of unhydrated binders in C-G2 paste compared to C-G1 paste. This is related to a lower degree of hydration of C-G2 paste compared to C-G1 paste at the age of 24 h. The results of the isothermal calorimetry, setting time measurement and microscopy all point to a delay in the hydration and solid skeleton development at early age in C-G2 paste compared to C-G1 paste. 

### 3.4. Desorption of SAPs in Pastes 

**Figure 5:**
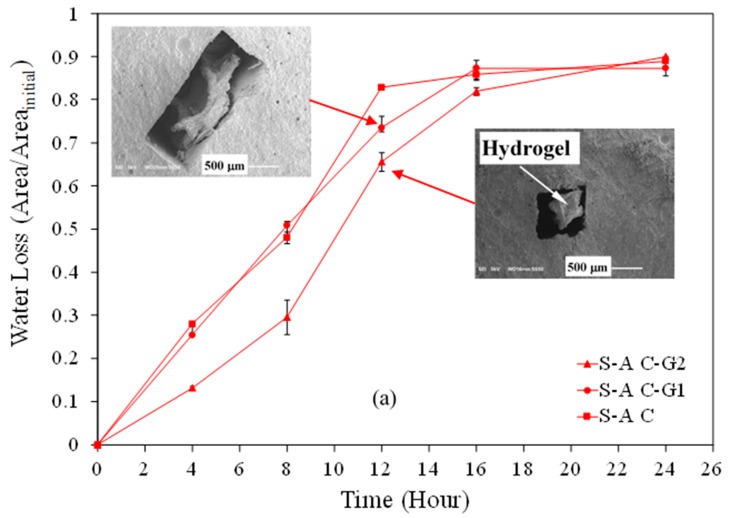

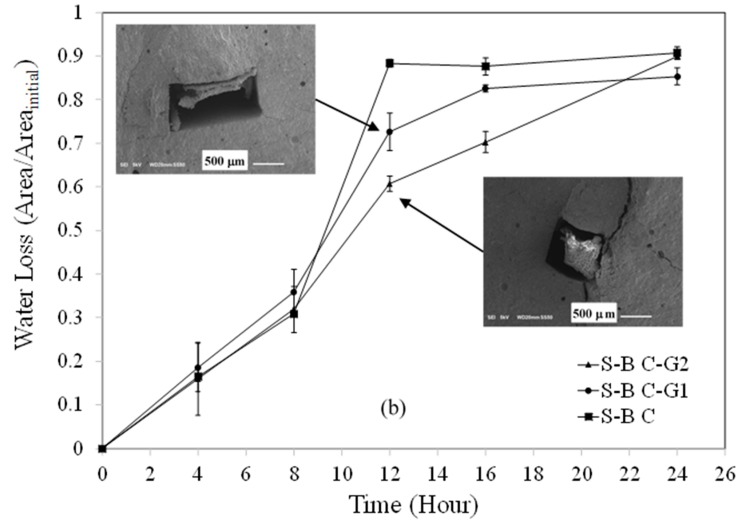
Water loss of (**a**) S-A and (**b**) S-B embedded in different pastes. Micrographs of embedded SAPs at the age of 12 h in C-G1 and C-G2 pastes are presented.

The desorption of SAP microstrips embedded in C, C-G1, and C-G2 pastes is shown in Figure 5a,b. The data corresponding to S-A C and S-B C are reproduced from data in [81]. Micrographs depicting the morphology change and desorption of S-A and S-B in C-G2 and C-G1 at the age of 12 h are shown in these figures. Both SAPs desorbed continuously until 24 h, after which time no significant change in desorption could be observed. It is noted that S-B seemed to have a higher desorption rate between 8 h and 12 h compared to S-A. This could be attributed to more effective capillary forces on S-B than S-A. Prior investigations showed a stronger contact between S-B and a cementitious matrix than S-A and a cementitious matrix [22]. The contact between the SAPs and the porous cementitious matrix can affect the desorption, as the influence of capillary forces can be realized when there is a contact between the SAPs and the porous cementitious matrix. Note the general delay in the desorption of S-A and S-B in C-G2 paste compared to C and C-G1 pastes. An explanation for this delay in desorption can be sought in the hydration and microstructure evolution of the pastes. As discussed previously, the setting time measurement, isothermal calorimetry, and SEM examination indicated a delay in hydration and solid skeleton development in C-G2 paste compared to the other two pastes. Due to a delay in relative humidity reduction and microstructure densification at early age in C-G2 paste, the SAPs experienced the effect of the capillary forces in C-G2 paste later than in C and C-G1 pastes, and as a result, the desorption occurred at a lower rate in C-G2 paste than in the other two pastes. These observations will provide valuable insight into the process of desorption of SAPs in cementitious materials, which will be necessary for the accurate design of cementitious materials/SAPs composite systems. 

### 3.5. Autogenous Shrinkage

**Figure 6:**
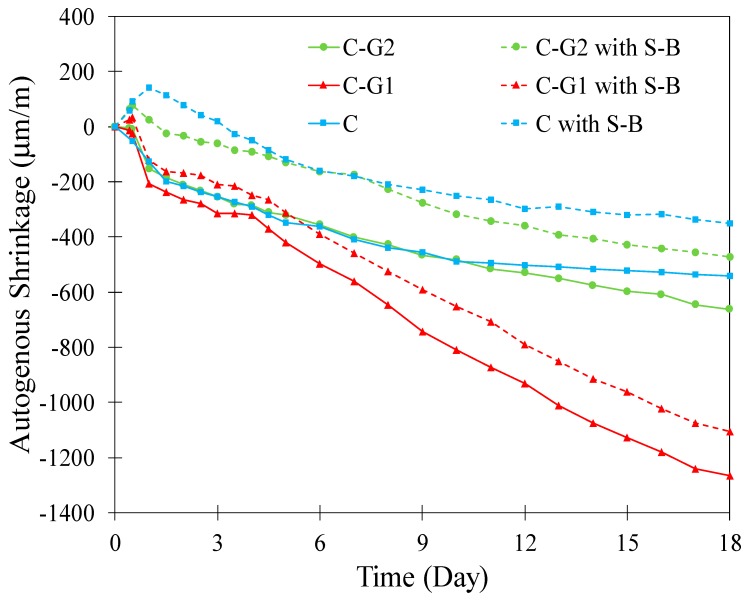
Autogenous shrinkage of different pastes with and without S-B.

The autogenous shrinkage of different pastes with and without S-B is shown in Figure 6. All pastes without SAPs showed a similar shrinkage up to 5 days; however, after this age, there is a sharp increase in shrinkage in C-G1 paste compared to C paste. In the case of C-G2 paste, an increase in shrinkage occurred at a later age (10 days) compared to C paste. The observed shrinkage in paste with glass powders could be due to the pozzolanic reaction, which is not active at early age and becomes active after formation of Ca(OH)_2_. The pozzolanic reaction contributes to microstructure densification, and as a result, increases the capillary forces and autogenous shrinkage in the pastes [62]. Pastes with S-B showed a general reduction in shrinkage compared to pastes without S-B; however, this reduction was smaller in the paste with G2 and more notably in the paste with G1, compared to the neat cement paste. Additionally, note that the autogenous shrinkage curves of each paste with and without S-B followed a parallel trend as seen from Figure 6. A small expansion after the setting time in C with S-B is observed; such expansions have also been documented in prior studies. Reabsorption of bleeding water and crystallization pressure of Ca(OH)_2_ formation have been suggested as possible reasons for this expansion [36,62,91].

## 4. Conclusions

The absorption and desorption behavior of SAPs in cement mixtures with and without glass powders as supplementary cementitious materials were investigated. The following conclusions are from the results of this study.

- SAP absorption can be affected by addition of supplementary cementitious materials; generally, an increase in SAP absorption was observed when glass powders were used.
- The absorption behavior depended on the chemical compositions of SAPs; an increase in acrylic acid/acrylamide decreased absorption in SAPs.
- The results of isothermal calorimetry, setting time measurement and microscopy all indicated a delay in hydration and solid skeleton development at early age in the paste with G2 compared to the paste with G1.
- The SAPs exhibited a lower desorption rate in the paste with G2 than in the other two pastes; this can be attributed to a delay in relative humidity reduction and densification of microstructure, which are the driving factors for SAP desorption.
- The paste with G1 and G2 showed an increase in autogenous shrinkage, compared to the neat cement paste after approximately five days and 10 days, respectively. This is most likely due to the pozzolanic reaction, which results in finer pore structure.
- The addition of SAP-B reduced autogenous shrinkage in the pastes due to internal curing, and the reduction was more in neat cement paste than in the pastes with G1 and G2.

## Figures and Tables

**Table 1:** Compositions of the superabsorbent polymer (SAP).

SAP Designation	Acrylamide (g)	Acrylic Acid (g)	MBA (Cross-linker) (g)	Ammonium Persulfate (g)	Sodium Hydroxide (g)	Distilled Water (g)
S-A	5	5	0.025	0.064	0.675	50
S-B	9	1	0.025	0.064	0.135	50

**Table 2:** Chemical compositions of cement, G1, and G2.

Composition (%)	Cement	G1	G2
SiO_2_	20.6	63.3	57.5
Al_2_O_3_	4.8	6.4	12.7
Fe_2_O_3_	3.5	0.31	0.06
CaO	64	17.1	22.7
MgO	0.9	4.5	3.6
Na_2_O	0.1	6.1	0.62
K_2_O	0.3	0.07	0.06
SO_3_	3.4	0.19	0.22

**Table 3:** Mix design of pastes and slurries.

Paste Designation	Water/Binder (Paste)	Water/Binder (Slurry)	Cement Replacement	Superplasticizer Percentage (per Binder Mass)
C	0.3	2	0%	0.5%
C-G1	0.3	2	40%	0.5%
C-G2	0.3	2	40%	0.5%

**Table 4:** Flow values and percentages of S-A and S-B in different pastes obtained from the flow test.

Paste Designation	S-A (% per Binder Mass)	S-B (% per Binder Mass)	Flow Value (cm)
C	0.42	0.17	20
C-G1	0.13	0.05	20
C-G2	0.33	0.08	20

**Table 5:** Setting times of C, C-G1, and C-G2 pastes.

Paste Designation	Initial Setting Time (min)	Final Setting Time (min)
C	345	495
C-G1	200	495
C-G2	525	705
